# Novel Computing Technologies for Bioinformatics and Cheminformatics

**DOI:** 10.1155/2014/392150

**Published:** 2014-12-28

**Authors:** Chuan Yi Tang, Che-Lun Hung, Ching-Hsien Hsu, Huiru Zheng, Chun-Yuan Lin

**Affiliations:** ^1^Department of Computer Science and Information Engineering, Providence University, Taichung 433, Taiwan; ^2^Department of Computer Science and Communication Engineering, Providence University, Taichung 433, Taiwan; ^3^Department of Computer Science and Information Engineering, Chung Hua University, Hsinchu 30012, Taiwan; ^4^School of Computing and Mathematics, Computer Science Research Institute, University of Ulster, Jordanstown BT370QB, UK; ^5^Department of Computer Science and Information Engineering, Chang Gung University, Taoyuan 333, Taiwan

## 1. Introduction

In the past, many computing technologies have been proposed and utilized to accelerate biologists/chemists to analyze biological and chemical data, such as homology detection, evolutionary analysis, function prediction, computer-aided drug design, and cheminformatics. Leveraging a power of these technologies, a lot of tools and services are valuable for biologists/chemists to efficiently analyze large-scale and complicated data. However, today's data is being generated and collected at an incredible scale, the buzzword “big data.” For instance, an individual laboratory can generate terabase scales of DNA and RNA sequencing data within a day by next-generation sequencing technologies. It is difficult to manage and process big biological and chemical data using conventional methods due to not only their size but also their complexity. It requires entirely different thoughts, while the major obstacle could be the complexity, size, or integration of various data sources. These barriers spur the revolutions of both storage and computing technologies whereby the developed tool and service can be highly scalable, totally reliable, more elastic, and so on.

Therefore, the computing technologies required to maintain, process, and integrate the large amounts of data are beyond the reach of small laboratories and introduce serious challenges even for large institutes. Success at the bioinformatics and cheminformatics fields will heavily rely on an ability to explain these large-scale and great diversification data, which encourage biologists/chemists to adopt novel computing technologies. The research papers selected for this special issue represent recent progresses in the aspects, including theoretical studies, practical applications, novel strategies and framework, high performance computing technologies, method and algorithm improvement, and review. All of these papers not only provide novel ideas and state of-the-art technologies in the field but also stimulate future research for Bioinformatics and Cheminformatics.

## 2. Large-Scale Biomedical Analysis

Recent progress in high-throughput instrumentations has led to an astonishing growth in both volume and complexity of biomedical data collected from various sources. The planet-size data brings serious challenges to the storage and computing technologies. The paper by Y.-C. Lin et al. entitled “*Enabling large-scale biomedical analysis in the cloud*” indicates the coming age of sharp data growth and increasing data diversification is a major challenge for biomedical research in the postgenome era. Cloud computing is an alternative to crack the nut because it gives concurrent consideration to enable storage and massive computing on large-scale data. Developing cloud-based biomedical applications can integrate the vast amount of diversification data in one place and analyze them on a continuous basis. This would make a significant breakthrough to launch a high quality healthcare. This review paper briefly introduces the data intensive computing system and summarizes existing cloud-based resources in bioinformatics. These developments and applications would facilitate biomedical research to make the vast amount of diversification data meaningful and usable.

With the rapid growth of next generation sequencing technologies, more and more data have been discovered and published. To analyze such huge data, the computational performance is an important issue. The paper by C.-L. Hung and G.-J. Hua entitled “*Local alignment tool based on hadoop framework and GPU architecture*” combines two different heterogeneous architectures, software architecture-Hadoop framework and hardware architecture-GPU, to develop a high performance cloud computing service, called Cloud-BLASTP, for protein sequence alignment. Cloud-BLASTP takes advantage of high performance, availability, reliability, and scalability. Cloud-BLASTP guarantees that all submitted jobs are properly completed, even when running job on an individual node or mapper experience failure. The performance experiment shows that Cloud-BLASTP is faster than GPU-BLASTP and is desirable for biologists to investigate the protein structure and function analysis by comparing large protein database under reasonable time constraints.

Organ segmentation is a crucial step prior to computer-aided diagnosis, since it is fundamental for further medical image processing such as cancer detection, lesion recognition, and three-dimensional visualization. However, organ extraction is considered as a challenge task due to huge shape variations, heterogeneous intensity distribution, and low contrast of CT image. The paper by H. Jiang et al. entitled “*A priori knowledge and probability density based segmentation method for medical CT image sequences*” briefly introduces a novel segmentation strategy for CT images sequences. In their strategy, a priori knowledge is effectively used to guide the determination of objects and a modified distance regularization level set method can accurately extract actual contour of object in a short time. Their proposed method is compared to other seven state-of-the-art medical image segmentation methods, GAC, C-V, SPLS, HLS, SDLS, CCRG, and IVLS, on abdominal CT image sequences datasets. The evaluated results demonstrate their method performs better and has the potential for segmentation in CT image sequences.

## 3. Novel Strategies for Drug Design

Quantitative structure-activity relationships (QSAR) is a widely adapted computational method that correlates the structural properties of compounds with their biological activities, such as the affinity between the ligand and protein and the toxicity of existing/hypothetical molecules. Recently, the prediction quality using the QSAR method was improved by considering the three-dimensional structure (3D-QSAR) of targeted inhibitors. The paper by C.-Y. Lin and Y.-L. Wang entitled “*Novel design strategy for checkpoint kinase 2 inhibitors using pharmacophore modeling, combinatorial fusion, and virtual screening*” proposes a novel design strategy for drug design by applying combinatorial fusion into pharmacophore hypotheses and virtual screening techniques. They first used 3D-QSAR study to build pharmacophore hypotheses for Chk2 inhibitors by HypoGen Best, Fast, and Caesar algorithms, respectively. Then, they used the combinatorial fusion to select and combine prediction results for improving the predictive accuracy in biological activities of inhibitors. Finally, all of feasible compounds in NCI database were selected by using ligand-based virtual screening.

Aptamers are an interesting alternative to antibodies in pharmaceutics and biosensorics, because they are able to bind to a multitude of possible target molecules with high affinity. Therefore, the process of finding such aptamers, which is commonly a SELEX screening process, becomes crucial. The standard SELEX procedure schedules the validation of certain found aptamers via binding experiments, which is not leading to any detailed specification of the aptamer enrichment during the screening. The paper by R. Beier et al. entitled “*New strategies for evaluation and analysis of SELEX experiments*” uses sequence information gathered by next generation sequencing techniques on SELEX experiments. They propose a motif search algorithm which helps to describe the aptamers enrichment in more detail. The extensive characterization of target and binding aptamers may later reveal a functional connection between these molecules, which can be modeled and used to optimize future SELEX runs in case of the generation of target-specific starting libraries.

## 4. Computational Genomics

Human and other primate genomes consist of segmental duplications due to fixation of copy number variations. Structure of these duplications within the human genome has been shown to be a complex mosaic composed of juxtaposed subunits, called duplicons. These duplicons are difficult to be uncovered from the mosaic repeat structure. In addition, the distribution and evolution of duplicons among primates are still poorly investigated. The paper by T.-J. Chuang et al. entitled “*A novel framework for the identification and analysis of duplicons between human and chimpanzee*” develops a statistical framework for discovering duplicons via integration of a Hidden Markov Model (HMM) and a permutation test. Their experimental results indicate that the mosaic structure composed of duplicons is common in copy number variations and segmental duplications of both human and chimpanzee. Gene ontology analysis, hierarchical clustering, and phylogenetic analysis of duplicons also were used in their work and then suggested that most copy number variations/segmental duplications share common duplication ancestry.

Due to the availability of abundant genomic resources, rice has become a model species for the genomic study. Bacterial blight, caused by* Xanthomonas oryzae* pv. oryzae (Xoo), is a worldwide devastating disease, and bacterial blight resistance genes have been cloned by a map-based cloning approach. It is important to find a more effective way to locate vital resistant genes. The text mining strategy represents another effective way to improve the efficiency of gene discovery. The paper by J. Xia et al. entitled “*Gene prioritization of resistant rice gene against Xanthomas oryzae pv. oryzae by using text mining technologies*” proposes a hybrid strategy to enhance gene prioritization by combining text mining technologies with a sequence-based approach. Their scheme consists of two sieves, the text-mining sieve and the classifier sieve. The text-mining sieve is to screen candidate gene according to the important phrase evaluation through TF ∗ IDF and voting scheme. The classifier sieve is a built-in classifier based on chaos games representation. Their experiment results show that the hybrid strategy achieves enhanced gene prioritization.

## 5. Computational Systems Biology

Genome-wide association studies for the analysis of gene-gene interaction are important fields for detecting the effects of cancer and disease. Such studies usually entail the collection of a vast number of samples and SNPs selected from several related genes of disease in order to identify the association amongst genes. A method for searching high-order interactions is needed to determine the potential association between several loci. Statistical methods are widely used to search for a good model of gene-gene interaction for disease analysis; however, the huge numbers of potential combinations of SNP genotypes limit the use of statistical methods for analysing high-order interaction. It remains a challenge to find an available high-order model of gene-gene interaction. The paper by C.-H. Yang et al. entitled “*Double-bottom chaotic map particle swarm optimization based on chi-square test to determine gene-gene interactions*” presents an improved particle swarm optimization with double-bottom chaotic maps (DBM-PSO) to assist statistical methods in the analysis of associated variations to disease susceptibility. Analysis results supported that the DBM-PSO is a robust and precise algorithm, and it can identify good models and provide higher chi-square values than conventional PSO.

Using microarray technology combined with computational analysis is one of the most efficient and cost-effective methods for studying cancer. Most studies focus primarily on identifying differential gene expressions between conditions, for discovering the major factors that cause diseases. Previous studies have not identified the correlations of differential gene expression between conditions; crucial but abnormal regulations that cause diseases might have been disregarded. The paper by T.-H. Chang et al. entitled “*A novel approach for discovering condition-specific correlations of gene expressions within biological pathways by using cloud computing technology*” proposes a novel approach for discovering the condition-specific correlations of gene expressions within biological pathways. An Apache Hadoop cloud computing platform was implemented to reduce the time for analyzing gene expression correlations. The experimental results showed that breast cancer recurrence might be highly associated with the abnormal regulations of these gene pairs, rather than with their individual expression levels. Their proposed method was computationally efficient and reliable for identifying meaningful biological regulation patterns between conditions.

## 6. Conclusions

All of the above papers address either cloud computing service or novel strategies for large-scale biomedical analysis and drug design. They also develop related method and approach improvements in applications of computational genomics and systems biology. Honorably, this special issue serves as a landmark source for education, information, and reference to professors, researchers, and graduate students interested in updating their knowledge about or active in biomedical analysis, drug design, computational genomics, and systems biology.

